# (*E*)-*N*-Butyl-3-(3,4-dihy­droxy­phen­yl)acryl­amide hemihydrate

**DOI:** 10.1107/S1600536812005570

**Published:** 2012-02-17

**Authors:** Yan Han, Mi-Hua Hao

**Affiliations:** a College of Chemistry and Chemical Engineering, Xinxiang University, Xinxiang, Henan 453003, People’s Republic of China

## Abstract

In the title compound, C_13_H_17_NO_3_·0.5H_2_O, a new caffeic acid amide derivative, the solvent water mol­ecule lies on a twofold axis and the terminal ethyl group appears disordered with occupancy factors of 0.525 (6) and 0.475 (6). The benzene ring makes an angle of 17.3 (2)° with the C=C—C—O linker. The presence of an ethyl­enic spacer in the caffeic acid amide mol­ecule allows the formation of a conjugated system, strongly stabilized through π-electron delocalization. The C=C double bond in the linker is *trans*, similar to those previously reported in caffeic esters. The crystal is stabilized by O—H⋯O, N—H⋯O and C—H⋯O hydrogen bonds. The mol­ecules of the caffeic acid amide form a supermolecular planar structure through O—H⋯O hydrogen bonds between a hy­droxy group of one caffeic acid mol­ecule and a carbonyl O atom of another. These planes inter­act *via* C—H⋯O, O—H⋯O and N—H⋯O hydrogen bonds to form a three-dimensional network.

## Related literature

For phenolic acid compounds used in biology and medicine, see: Altuğ *et al.* (2008 ▶). For synthetic work on the similar compounds, see: Bylov *et al.* (1999 ▶). For compounds with similar properties, see: Son & Lewis (2002 ▶); Menezes *et al.* (2001 ▶); Lee *et al.* (2005 ▶). For the structure analysis of a similar compound, see: Xia *et al.* (2008 ▶).
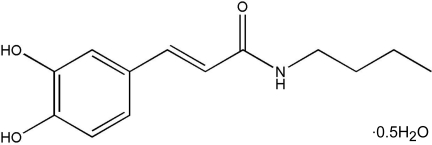


## Experimental

### 

#### Crystal data

C_13_H_17_NO_3_·0.5H_2_O
*M* *_r_* = 244.29
Monoclinic, 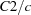

*a* = 12.860 (7) Å
*b* = 14.928 (8) Å
*c* = 15.015 (11) Å
β = 113.967 (6)°
*V* = 2634 (3) Å^3^
*Z* = 8
Mo *K*α radiation
μ = 0.09 mm^−1^
*T* = 296 K
0.28 × 0.22 × 0.19 mm

#### Data collection

Bruker SMART APEX CCD diffractometer
Absorption correction: multi-scan (*SADABS*; Sheldrick, 2008 ▶) *T*_min_ = 0.977, *T*_max_ = 0.983
7822 measured reflections
2581 independent reflections
1895 reflections with *I* > 2σ(*I*)
*R*_int_ = 0.025

#### Refinement

*R*[*F* ^2^ > 2σ(*F* ^2^)] = 0.042
*wR*(*F* ^2^) = 0.120
*S* = 1.04
2581 reflections
180 parameters
H-atom parameters constrained
Δρ_max_ = 0.18 e Å^−3^
Δρ_min_ = −0.21 e Å^−3^

### 

Data collection: *SMART* (Bruker, 2008 ▶); cell refinement: *SAINT* (Bruker, 2008 ▶); data reduction: *SAINT*; program(s) used to solve structure: *SHELXS97* (Sheldrick, 2008 ▶); program(s) used to refine structure: *SHELXL97* (Sheldrick, 2008 ▶); molecular graphics: *SHELXTL* (Sheldrick, 2008 ▶); software used to prepare material for publication: *SHELXTL*.

## Supplementary Material

Crystal structure: contains datablock(s) I, global. DOI: 10.1107/S1600536812005570/bg2440sup1.cif

Structure factors: contains datablock(s) I. DOI: 10.1107/S1600536812005570/bg2440Isup2.hkl

Supplementary material file. DOI: 10.1107/S1600536812005570/bg2440Isup3.cdx

Supplementary material file. DOI: 10.1107/S1600536812005570/bg2440Isup4.cml

Additional supplementary materials:  crystallographic information; 3D view; checkCIF report

## Figures and Tables

**Table 1:** Hydrogen-bond geometry (Å, °)

*D*—H⋯*A*	*D*—H	H⋯*A*	*D*⋯*A*	*D*—H⋯*A*
O4—H4*A*⋯O3^i^	0.85	1.91	2.7402 (19)	165
O4—H4*B*⋯O3^ii^	0.85	1.91	2.7402 (19)	165
N1—H1*B*⋯O2^iii^	0.86	2.29	3.129 (2)	165
N1—H1*B*⋯O1^iii^	0.86	2.58	3.143 (3)	124
O1—H1*A*⋯O3^iv^	0.82	1.94	2.7378 (19)	162
O2—H2*A*⋯O4^v^	0.82	2.00	2.8217 (18)	177
C7—H7⋯O3	0.93	2.48	2.837 (2)	103
C8—H8⋯O2^iii^	0.93	2.55	3.330 (2)	142

Symmetry codes: (i) 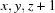; (ii) 
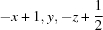
; (iii) 
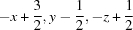
; (iv) 
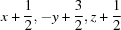
; (v) 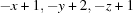.

**Fig. 1.**
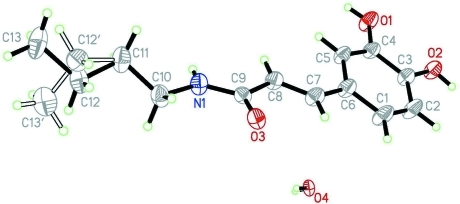
View of the molecular structure of (E)-N-butyl-3-(3,4-dihydroxyphenyl)acrylamide monohydrate with displacement ellipsoids at the 30% probability level. In open bonds, the minor part of the disordered tail.

**Fig. 2.**
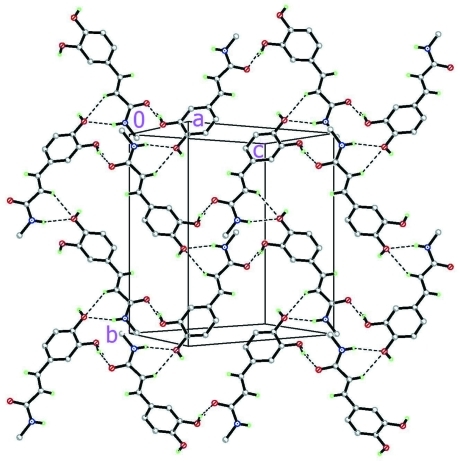
The two-dimensional plane formed by the hydrogen bonds of the molecules (Dashed lines represent hydrogen bonds, some of the H and C atoms have been omitted)

## supplementary crystallographic information

### Comment

Phenolic acids and their derivatives (esters, amides) are widely distributed in plants and can be present in considerable amounts in the human diet, which attract much attention in biology and medicine (Altuğ *et al.*, 2008). Phenolic acid amides, is a large class of organic compounds formed by the condensation reaction of aromatic acid and amine (Bylov *et al.*, 1999). The –CO—NH– backbone makes amides have the pharmacological functionality such as anti-proliferative, antiviral, antimalarial, general anesthetics and antimicrobials. Therefore, more and more phenolic acid amides have already been researched purposively and developed as potential anti-proliferative, antiviral, antimalarial, and antimicrobials drugs in recent years (Son & Lewis, 2002; Menezes *et al.*, 2001; Lee *et al.*, 2005). So in this paper, we synthesized and report on the structure of a new caffeic acid amide derivative, (*E*)-*N*-butyl-3-(3,4-dihydroxyphenyl)acrylamide monohydrate.

In the title compound (Fig. 1), all values of the geometric parameters are normal, the terminal C12—C13 group being disorderd with occupation factors of 0.525/0.475 (6) for both moieties. The benzene ring is planar within experimental observation (r.m.s. deviation: 0.005Å) and it makes an angle of 17.3 (2)° to the (C7—C8—C9—O3) linker. In the case of caffeic amide, the presence of an ethylenic spacer allows the formation of a conjugated system, strongly stabilized through π-electron delocalization. The C7═C8 bond is a *trans*-double bond, the same as in caffeic esters reported before (Xia *et al.*, 2008).

The crystal is stabilized by intermolecular O—H···O, N—H···O, O—H···N and C—H···O hydrogen bonds (Fig. 2). The molecules of the caffeic acid amide form a supermolecule plane structure through O—H···O hydrogen bonds between a hydroxyl of a caffeic acid and carbonyl O atom from another caffeic acid molecule. The supermolecule plane interacts with the another plane with C—H···O, O—H···O, N—H···O and O—H···N hydrogen bonds to form a three-dimensional network.

### Experimental

To a stirred THF solution (15 ml) of commercial caffeic acid (0.179 g, 1 mmol) was added DCC (1.5 mmol) in THF (5 ml) at 0°C. The reaction mixture was stirred for 2 h at 0°C and *n*-butylamine (0.088 g, 1.2 mmol) was added for reacting overnight at room temperature, and then the precipitated dicyclohexylurea was filtered and washed with tetrahydrofuran. The combined filtrates were evaporated, the residue was extracted with EtOAc, and the extract was washed with 1 N NaHCO_3_, H_2_O, and 1 N HCl, dried over Na_2_SO_4_, and evaporated. The title compound was obtained by elution of the column with 25% petrole:EtOAc and recrystallized to give the pale yellow block crystal.

### Refinement

All the H atoms were seen in a difference map but repositioned geometrically (C—H = 0.93–0.97, N—H = 0.86 and O—H = 0.85 Å) and refined as riding with *U*_iso_ = 1.2Ueq. The C12—C13 pair is disordered into two moieties with occupancies of 0.525/0.475 (6).

### Crystal data

**Table:**

C_13_H_17_NO_3_·0.5H_2_O	*F*(000) = 1048
*M**_r_* = 244.29	*D*_x_ = 1.232 Mg m^−^^3^
Monoclinic, *C*2/*c*	Mo *K*α radiation, λ = 0.71073 Å
Hall symbol: -C 2yc	Cell parameters from 2581 reflections
*a* = 12.860 (7) Å	θ = 2.2–26.0°
*b* = 14.928 (8) Å	µ = 0.09 mm^−^^1^
*c* = 15.015 (11) Å	*T* = 296 K
β = 113.967 (6)°	Block, yellow
*V* = 2634 (3) Å^3^	0.28 × 0.22 × 0.19 mm
*Z* = 8	

### Data collection

**Table:**

Bruker SMART APEX CCD diffractometer	2581 independent reflections
Radiation source: fine-focus sealed tube	1895 reflections with *I* > 2σ(*I*)
Graphite monochromator	*R*_int_ = 0.025
φ and ω scans	θ_max_ = 26.0°, θ_min_ = 2.2°
Absorption correction: multi-scan (*SADABS*; Sheldrick, 2008)	*h* = −15→15
*T*_min_ = 0.977, *T*_max_ = 0.983	*k* = −18→15
7822 measured reflections	*l* = −18→18

### Refinement

**Table:**

Refinement on *F*^2^	Primary atom site location: structure-invariant direct methods
Least-squares matrix: full	Secondary atom site location: difference Fourier map
*R*[*F*^2^ > 2σ(*F*^2^)] = 0.042	Hydrogen site location: inferred from neighbouring sites
*wR*(*F*^2^) = 0.120	H-atom parameters constrained
*S* = 1.04	*w* = 1/[σ^2^(*F*_o_^2^) + (0.0532*P*)^2^ + 1.0544*P*] where *P* = (*F*_o_^2^ + 2*F*_c_^2^)/3
2581 reflections	(Δ/σ)_max_ < 0.001
180 parameters	Δρ_max_ = 0.18 e Å^−^^3^
0 restraints	Δρ_min_ = −0.21 e Å^−^^3^

### Special details

**Table:**

Geometry. All e.s.d.'s (except the e.s.d. in the dihedral angle between two l.s. planes) are estimated using the full covariance matrix. The cell e.s.d.'s are taken into account individually in the estimation of e.s.d.'s in distances, angles and torsion angles; correlations between e.s.d.'s in cell parameters are only used when they are defined by crystal symmetry. An approximate (isotropic) treatment of cell e.s.d.'s is used for estimating e.s.d.'s involving l.s. planes.
Refinement. Refinement of *F*^2^ against ALL reflections. The weighted *R*-factor *wR* and goodness of fit *S* are based on *F*^2^, conventional *R*-factors *R* are based on *F*, with *F* set to zero for negative *F*^2^. The threshold expression of *F*^2^ > σ(*F*^2^) is used only for calculating *R*-factors(gt) *etc*. and is not relevant to the choice of reflections for refinement. *R*-factors based on *F*^2^ are statistically about twice as large as those based on *F*, and *R*-factors based on ALL data will be even larger. The terminal C13 and methylene C12 have been treated disorderd.

### Fractional atomic coordinates and isotropic or equivalent isotropic displacement parameters (Å^2^)

**Table:**

	*x*	*y*	*z*	*U*_iso_*/*U*_eq_	Occ. (<1)
O4	0.5000	0.79934 (9)	0.7500	0.0471 (4)	
H4A	0.5047	0.7659	0.7973	0.057*	0.50
H4B	0.4953	0.7659	0.7027	0.057*	0.50
N1	0.63571 (12)	0.56741 (9)	−0.01730 (9)	0.0517 (4)	
H1B	0.6878	0.5587	0.0401	0.062*	
O1	0.83466 (10)	0.95089 (8)	0.33417 (8)	0.0653 (4)	
H1A	0.8785	0.9099	0.3375	0.098*	
O2	0.67900 (9)	1.07499 (8)	0.30337 (8)	0.0558 (3)	
H2A	0.6254	1.1101	0.2869	0.084*	
O3	0.51295 (9)	0.66595 (7)	−0.12054 (7)	0.0493 (3)	
C1	0.53067 (15)	0.93015 (13)	0.09233 (13)	0.0656 (6)	
H1	0.4611	0.9268	0.0388	0.079*	
C2	0.55145 (15)	1.00043 (13)	0.15684 (13)	0.0638 (5)	
H2	0.4962	1.0443	0.1459	0.077*	
C3	0.65313 (13)	1.00632 (11)	0.23735 (11)	0.0491 (4)	
C4	0.73642 (13)	0.94036 (11)	0.25296 (11)	0.0479 (4)	
C5	0.71557 (13)	0.87094 (11)	0.18753 (11)	0.0498 (4)	
H5	0.7713	0.8277	0.1977	0.060*	
C6	0.61164 (14)	0.86440 (12)	0.10578 (11)	0.0522 (4)	
C7	0.58305 (13)	0.79218 (12)	0.03400 (12)	0.0520 (4)	
H7	0.5189	0.8017	−0.0233	0.062*	
C8	0.63569 (13)	0.71565 (11)	0.03940 (11)	0.0483 (4)	
H8	0.7026	0.7038	0.0935	0.058*	
C9	0.59066 (12)	0.64825 (10)	−0.03873 (10)	0.0410 (4)	
C10	0.60274 (16)	0.49214 (12)	−0.08466 (13)	0.0608 (5)	
H10A	0.5750	0.5145	−0.1509	0.073*	
H10B	0.5411	0.4598	−0.0776	0.073*	
C11	0.69973 (18)	0.42951 (12)	−0.06711 (14)	0.0678 (5)	
H11A	0.7326	0.4095	0.0002	0.081*	
H11B	0.7585	0.4582	−0.0819	0.081*	
C12	0.6467 (8)	0.3505 (6)	−0.1365 (8)	0.077 (2)	0.525 (6)
H12A	0.5909	0.3206	−0.1188	0.093*	0.525 (6)
H12B	0.6088	0.3720	−0.2029	0.093*	0.525 (6)
C13	0.7411 (5)	0.2857 (3)	−0.1286 (4)	0.097 (2)	0.525 (6)
H13A	0.7933	0.3148	−0.1503	0.146*	0.525 (6)
H13B	0.7089	0.2342	−0.1686	0.146*	0.525 (6)
H13C	0.7808	0.2673	−0.0620	0.146*	0.525 (6)
C12'	0.6881 (7)	0.3529 (8)	−0.1425 (9)	0.073 (2)	0.475 (6)
H12C	0.6553	0.3766	−0.2083	0.088*	0.475 (6)
H12D	0.7624	0.3283	−0.1305	0.088*	0.475 (6)
C13'	0.6133 (5)	0.2818 (4)	−0.1313 (4)	0.098 (2)	0.475 (6)
H13D	0.6453	0.2599	−0.0655	0.147*	0.475 (6)
H13E	0.6074	0.2334	−0.1752	0.147*	0.475 (6)
H13F	0.5391	0.3061	−0.1458	0.147*	0.475 (6)

### Atomic displacement parameters (Å^2^)

**Table:**

	*U*^11^	*U*^22^	*U*^33^	*U*^12^	*U*^13^	*U*^23^
O4	0.0635 (10)	0.0324 (8)	0.0410 (8)	0.000	0.0166 (7)	0.000
N1	0.0572 (8)	0.0449 (8)	0.0358 (7)	0.0049 (6)	0.0011 (6)	0.0003 (6)
O1	0.0455 (6)	0.0695 (9)	0.0532 (7)	0.0148 (6)	−0.0085 (5)	−0.0213 (6)
O2	0.0493 (6)	0.0568 (7)	0.0478 (7)	0.0099 (5)	0.0059 (5)	−0.0159 (5)
O3	0.0523 (6)	0.0427 (7)	0.0359 (6)	−0.0050 (5)	0.0005 (5)	0.0034 (5)
C1	0.0442 (9)	0.0799 (14)	0.0484 (10)	0.0186 (9)	−0.0062 (8)	−0.0193 (9)
C2	0.0478 (9)	0.0726 (13)	0.0521 (10)	0.0236 (9)	0.0006 (8)	−0.0175 (9)
C3	0.0446 (8)	0.0536 (10)	0.0400 (8)	0.0058 (7)	0.0079 (7)	−0.0120 (7)
C4	0.0378 (8)	0.0562 (10)	0.0373 (8)	0.0061 (7)	0.0024 (6)	−0.0053 (7)
C5	0.0412 (8)	0.0523 (10)	0.0447 (9)	0.0120 (7)	0.0060 (7)	−0.0071 (7)
C6	0.0457 (9)	0.0580 (11)	0.0399 (9)	0.0090 (7)	0.0042 (7)	−0.0110 (8)
C7	0.0413 (8)	0.0614 (11)	0.0389 (8)	0.0057 (7)	0.0015 (7)	−0.0087 (7)
C8	0.0424 (8)	0.0521 (10)	0.0372 (8)	0.0028 (7)	0.0025 (7)	−0.0036 (7)
C9	0.0393 (7)	0.0435 (9)	0.0344 (8)	−0.0038 (6)	0.0092 (6)	0.0020 (6)
C10	0.0609 (11)	0.0474 (11)	0.0547 (10)	−0.0030 (8)	0.0033 (9)	−0.0042 (8)
C11	0.0828 (14)	0.0510 (11)	0.0556 (11)	0.0111 (10)	0.0138 (10)	0.0000 (9)
C12	0.096 (6)	0.048 (4)	0.090 (4)	−0.003 (4)	0.040 (4)	−0.018 (3)
C13	0.141 (5)	0.061 (3)	0.106 (4)	0.029 (3)	0.067 (3)	−0.009 (2)
C12'	0.076 (5)	0.066 (5)	0.089 (4)	−0.003 (4)	0.044 (4)	−0.008 (3)
C13'	0.113 (5)	0.074 (4)	0.109 (4)	−0.002 (3)	0.046 (4)	0.001 (3)

### Geometric parameters (Å, º)

**Table:**

O4—H4A	0.8501	C8—C9	1.474 (2)
O4—H4B	0.8501	C8—H8	0.9300
N1—C9	1.321 (2)	C10—C11	1.494 (3)
N1—C10	1.455 (2)	C10—H10A	0.9700
N1—H1B	0.8600	C10—H10B	0.9700
O1—C4	1.3624 (19)	C11—C12	1.537 (9)
O1—H1A	0.8200	C11—C12'	1.574 (11)
O2—C3	1.3699 (19)	C11—H11A	0.9700
O2—H2A	0.8200	C11—H11B	0.9700
O3—C9	1.2571 (18)	C12—C13	1.519 (10)
C1—C2	1.378 (2)	C12—H12A	0.9700
C1—C6	1.385 (2)	C12—H12B	0.9700
C1—H1	0.9300	C13—H13A	0.9600
C2—C3	1.377 (2)	C13—H13B	0.9600
C2—H2	0.9300	C13—H13C	0.9600
C3—C4	1.403 (2)	C12'—C13'	1.487 (11)
C4—C5	1.377 (2)	C12'—H12C	0.9700
C5—C6	1.403 (2)	C12'—H12D	0.9700
C5—H5	0.9300	C13'—H13D	0.9600
C6—C7	1.462 (2)	C13'—H13E	0.9600
C7—C8	1.314 (2)	C13'—H13F	0.9600
C7—H7	0.9300		
			
H4A—O4—H4B	108.0	N1—C10—C11	111.97 (15)
C9—N1—C10	124.15 (14)	N1—C10—H10A	109.2
C9—N1—H1B	117.9	C11—C10—H10A	109.2
C10—N1—H1B	117.9	N1—C10—H10B	109.2
C4—O1—H1A	109.5	C11—C10—H10B	109.2
C3—O2—H2A	109.5	H10A—C10—H10B	107.9
C2—C1—C6	121.15 (15)	C10—C11—C12	104.6 (4)
C2—C1—H1	119.4	C10—C11—C12'	120.0 (4)
C6—C1—H1	119.4	C10—C11—H11A	110.8
C3—C2—C1	120.63 (15)	C12—C11—H11A	110.8
C3—C2—H2	119.7	C12'—C11—H11A	113.6
C1—C2—H2	119.7	C10—C11—H11B	110.8
O2—C3—C2	123.23 (14)	C12—C11—H11B	110.8
O2—C3—C4	117.38 (14)	C12'—C11—H11B	90.6
C2—C3—C4	119.37 (15)	H11A—C11—H11B	108.9
O1—C4—C5	124.49 (14)	C13—C12—C11	108.4 (6)
O1—C4—C3	115.86 (14)	C13—C12—H12A	110.0
C5—C4—C3	119.65 (14)	C11—C12—H12A	110.0
C4—C5—C6	121.09 (14)	C13—C12—H12B	110.0
C4—C5—H5	119.5	C11—C12—H12B	110.0
C6—C5—H5	119.5	H12A—C12—H12B	108.4
C1—C6—C5	118.09 (15)	C13'—C12'—C11	108.3 (6)
C1—C6—C7	117.78 (15)	C13'—C12'—H12C	110.0
C5—C6—C7	124.13 (15)	C11—C12'—H12C	110.0
C8—C7—C6	128.82 (15)	C13'—C12'—H12D	110.0
C8—C7—H7	115.6	C11—C12'—H12D	110.0
C6—C7—H7	115.6	H12C—C12'—H12D	108.4
C7—C8—C9	121.24 (15)	C12'—C13'—H13D	109.5
C7—C8—H8	119.4	C12'—C13'—H13E	109.5
C9—C8—H8	119.4	H13D—C13'—H13E	109.5
O3—C9—N1	121.73 (14)	C12'—C13'—H13F	109.5
O3—C9—C8	122.27 (14)	H13D—C13'—H13F	109.5
N1—C9—C8	116.00 (13)	H13E—C13'—H13F	109.5
			
C6—C1—C2—C3	−0.9 (3)	C5—C6—C7—C8	12.7 (3)
C1—C2—C3—O2	179.32 (18)	C6—C7—C8—C9	176.70 (16)
C1—C2—C3—C4	0.6 (3)	C10—N1—C9—O3	1.0 (2)
O2—C3—C4—O1	1.2 (2)	C10—N1—C9—C8	−179.89 (15)
C2—C3—C4—O1	179.99 (17)	C7—C8—C9—O3	12.8 (2)
O2—C3—C4—C5	−178.59 (15)	C7—C8—C9—N1	−166.28 (16)
C2—C3—C4—C5	0.2 (3)	C9—N1—C10—C11	148.87 (17)
O1—C4—C5—C6	179.54 (17)	N1—C10—C11—C12	174.2 (4)
C3—C4—C5—C6	−0.7 (3)	N1—C10—C11—C12'	−169.8 (4)
C2—C1—C6—C5	0.4 (3)	C10—C11—C12—C13	176.3 (5)
C2—C1—C6—C7	−179.91 (18)	C12'—C11—C12—C13	37.4 (19)
C4—C5—C6—C1	0.4 (3)	C10—C11—C12'—C13'	−74.3 (8)
C4—C5—C6—C7	−179.25 (17)	C12—C11—C12'—C13'	−27.1 (17)
C1—C6—C7—C8	−166.91 (19)		

### Hydrogen-bond geometry (Å, º)

**Table:**

*D*—H···*A*	*D*—H	H···*A*	*D*···*A*	*D*—H···*A*
O4—H4*A*···O3^i^	0.85	1.91	2.7402 (19)	165
O4—H4*B*···O3^ii^	0.85	1.91	2.7402 (19)	165
N1—H1*B*···O2^iii^	0.86	2.29	3.129 (2)	165
N1—H1*B*···O1^iii^	0.86	2.58	3.143 (3)	124
O1—H1*A*···O3^iv^	0.82	1.94	2.7378 (19)	162
O2—H2*A*···O4^v^	0.82	2.00	2.8217 (18)	177
C7—H7···O3	0.93	2.48	2.837 (2)	103
C8—H8···O2^iii^	0.93	2.55	3.330 (2)	142

Symmetry codes: (i) *x*, *y*, *z*+1; (ii) −*x*+1, *y*, −*z*+1/2; (iii) −*x*+3/2, *y*−1/2, −*z*+1/2; (iv) *x*+1/2, −*y*+3/2, *z*+1/2; (v) −*x*+1, −*y*+2, −*z*+1.
